# Response to Biologic Drugs in Patients With Rheumatoid Arthritis and Antidrug Antibodies

**DOI:** 10.1001/jamanetworkopen.2023.23098

**Published:** 2023-07-12

**Authors:** Samuel Bitoun, Signe Hässler, David Ternant, Natacha Szely, Aude Gleizes, Christophe Richez, Martin Soubrier, Jérome Avouac, Olivier Brocq, Jérémie Sellam, Niek de Vries, Tom W. J. Huizinga, Elizabeth C. Jury, Jessica J. Manson, Claudia Mauri, Andrea Matucci, Salima Hacein Bey Abina, Denis Mulleman, Marc Pallardy, Philippe Broët, Xavier Mariette

**Affiliations:** 1 Rheumatology Department, Université Paris-Saclay, Institut National de la Santé et de la Recherche Médicale (INSERM) U1184, Hôpital Bicêtre, Assistance Publique–Hôpitaux de Paris (APHP), Fédération Hospitalo Universitaire Cancer and Autoimmunity Relationships, Paris, France; 2 Université Paris-Saclay, INSERM U1018, Centre de Recherche en Epidémiologie et Santé des Populations, Paris, France; 3 INSERM Unité Mixte de Recherche (UMR) 959, Immunology-Immunopathology-Immunotherapy (i3), Sorbonne Université, Paris, France; 4 APHP, Hôpital Pitié Salpêtrière, Biotherapy (CIC-BTi), Paris, France; 5 EA6295 NanoMedicines and NanoProbes, University of Tours, Tours, France; 6 Université Paris-Saclay, INSERM, Inflammation, Microbiome, Immunosurveillance, Faculty of Pharmacy, Paris, France; 7 Clinical Immunology Laboratory, Groupe Hospitalier Universitaire Paris-Saclay, Hôpital Bicêtre, APHP, Paris, France; 8 Université Paris Cité, Centre National de la Recherche Scientifique, INSERM, Unité des Technologies Chimiques et Biologiques pour la Santé, Paris, France; 9 Unité de Formation et de Recherche de Pharmacie, Université Paris-Saclay, Paris, France; 10 Rheumatology Department, Centre Hospitalier Universitaire (CHU) de Bordeaux-GH Pellegrin, Bordeaux, France; 11 Rheumatology Department, CHU Gabriel-Montpied, Clermont-Ferrand, France; 12 Rheumatology Department, Hôpital Cochin, APHP, Centre-Université de Paris Cité, Paris, France; 13 Rheumatology Department, Hôpital Princesse Grâce de Monaco, Monaco; 14 Rheumatology Department, Hôpital Saint-Antoine, APHP, Sorbonne University, INSERM UMR 938, Paris, France; 15 Rheumatology and Clinical Immunology, Amsterdam UMC, AMC University of Amsterdam, Amsterdam, the Netherlands; 16 Department of Rheumatology, Leiden University Medical Center, Leiden, the Netherlands; 17 Centre for Rheumatology Research, University College London, London, England; 18 Department of Rheumatology, University College London Hospital, London, England; 19 Division of Infection, Immunity and Transplantation, University College London, London, England; 20 Department of Immunology, Azienda Ospedaliero Universitaria Careggi, Florence, Italy

## Abstract

**Importance:**

There are conflicting data on the association of antidrug antibodies with response to biologic disease–modifying antirheumatic drugs (bDMARDs) in rheumatoid arthritis (RA).

**Objective:**

To analyze the association of antidrug antibodies with response to treatment for RA.

**Design, Setting, and Participants:**

This cohort study analyzed data from the ABI-RA (Anti-Biopharmaceutical Immunization: Prediction and Analysis of Clinical Relevance to Minimize the Risk of Immunization in Rheumatoid Arthritis Patients) multicentric, open, prospective study of patients with RA from 27 recruiting centers in 4 European countries (France, Italy, the Netherlands, and the UK). Eligible patients were 18 years or older, had RA diagnosis, and were initiating a new bDMARD. Recruitment spanned from March 3, 2014, to June 21, 2016. The study was completed in June 2018, and data were analyzed in June 2022.

**Exposures:**

Patients were treated with a new bDMARD: adalimumab, infliximab (grouped as anti–tumor necrosis factor [TNF] monoclonal antibodies [mAbs]), etanercept, tocilizumab, and rituximab according to the choice of the treating physician.

**Main Outcomes and Measures:**

The primary outcome was the association of antidrug antibody positivity with EULAR (European Alliance of Associations for Rheumatology; formerly, European League Against Rheumatism) response to treatment at month 12 assessed through univariate logistic regression. The secondary end points were the EULAR response at month 6 and at visits from month 6 to months 15 to 18 using generalized estimating equation models. Detection of antidrug antibody serum levels was performed at months 1, 3, 6, 12, and 15 to 18 using electrochemiluminescence (Meso Scale Discovery) and drug concentration for anti-TNF mAbs, and etanercept in the serum was measured using enzyme-linked immunosorbent assay.

**Results:**

Of the 254 patients recruited, 230 (mean [SD] age, 54.3 [13.7] years; 177 females [77.0%]) were analyzed. At month 12, antidrug antibody positivity was 38.2% in patients who were treated with anti-TNF mAbs, 6.1% with etanercept, 50.0% with rituximab, and 20.0% with tocilizumab. There was an inverse association between antidrug antibody positivity (odds ratio [OR], 0.19; 95% CI, 0.09-0.38; *P* < .001) directed against all biologic drugs and EULAR response at month 12. Analyzing all the visits starting at month 6 using generalized estimating equation models confirmed the inverse association between antidrug antibody positivity and EULAR response (OR, 0.35; 95% CI, 0.18-0.65; *P* < .001). A similar association was found for tocilizumab alone (OR, 0.18; 95% CI, 0.04-0.83; *P* = .03). In the multivariable analysis, antidrug antibodies, body mass index, and rheumatoid factor were independently inversely associated with response to treatment. There was a significantly higher drug concentration of anti-TNF mAbs in patients with antidrug antibody–negative vs antidrug antibody–positive status (mean difference, −9.6 [95% CI, −12.4 to −6.9] mg/L; *P* < 001). Drug concentrations of etanercept (mean difference, 0.70 [95% CI, 0.2-1.2] mg/L; *P* = .005) and adalimumab (mean difference, 1.8 [95% CI, 0.4-3.2] mg/L; *P* = .01) were lower in nonresponders vs responders. Methotrexate comedication at baseline was inversely associated with antidrug antibodies (OR, 0.50; 95% CI, 0.25-1.00; *P* = .05).

**Conclusions and Relevance:**

Results of this prospective cohort study suggest an association between antidrug antibodies and nonresponse to bDMARDs in patients with RA. Monitoring antidrug antibodies could be considered in the treatment of these patients, particularly nonresponders to biologic RA drugs.

## Key Points

**Question:**

Are antidrug antibodies associated with therapeutic response to biologic drugs in rheumatoid arthritis (RA)?

**Findings:**

In this cohort study of 230 patients with RA, the presence of antidrug antibodies was associated with a diminished response to biologic disease–modifying antirheumatic drugs. This association was also found for tocilizumab, an anti–interleukin 6 receptor therapeutic antibody.

**Meaning:**

Findings of this study suggest that antidrug antibodies are associated with nonresponse to biologic drugs in RA and can be monitored in the management of patients with RA, particularly nonresponders.

## Teaser

This cohort study evaluates the association between the presence of antidrug antibodies and response to biologic disease–modifying antirheumatic drugs, including adalimumab, infliximab, etanercept, tocilizumab, and rituximab, in adults with rheumatoid arthritis (RA).

## Introduction

Biologic drugs in rheumatology, frequently termed biologic disease–modifying antirheumatic drugs (bDMARDs), are recommended to be started as a second-line treatment for rheumatoid arthritis (RA). However, the retention rate is low, with only 40% to 60% of patients maintaining such treatment after 2 years.^1,2^ Among the factors associated with the ineffectiveness of these treatments are antidrug antibodies. These antibodies directed against biologic drugs might play a role in diminished drug concentration and loss of bDMARD effectiveness. The ABIRISK (Anti-Biopharmaceutical Immunization: Prediction and Analysis of Clinical Relevance to Minimize the Risk) study is a European Union Innovative Medicines Initiative aimed at identifying the risk factors for the generation of antidrug antibodies. ABIRISK includes prospective cohorts of patients with several autoimmune and inflammatory diseases, such as multiple sclerosis, RA, Crohn disease, and ulcerative colitis. A previous ABIRISK study found that the use of associated immunosuppressants and antibiotics was associated with a decreased risk of developing antidrug antibodies.^3^ Moreover in patients with RA, methotrexate comedication was associated with fewer antidrug antibodies.^4,5,6^

There is still debate regarding the adverse clinical implications of antidrug antibodies for the response to treatment, which has been clearly demonstrated only for adalimumab, an anti–tumor necrosis factor (TNF) monoclonal antibody (mAb).^7^ Recent publications reported that monitoring the drug concentration (which reflects the presence of antidrug antibodies) of infliximab, another anti-TNF mAb, affected the response only for maintenance but not during the induction period.^8,9^ Prospective data are scarce on the association of other bDMARDs with clinical response.^10^ Using ABI-RA (Anti-Biopharmaceutical Immunization: Prediction and Analysis of Clinical Relevance to Minimize the Risk of Immunization in Rheumatoid Arthritis Patients), a prospective ABIRISK study in RA, we aimed to analyze the association of antidrug antibodies (against TNF inhibitors, anti-CD20 mAbs [rituximab], and anti–interluekin 6 receptor mAbs [tocilizumab]) with response to treatment.

## Methods

This cohort study was restricted to analyzing data from the ABI-RA multicenter prospective study of patients with RA (NCT02116504). The study was approved by the Comité de Protection des Personnes Ile de France VII for France; the Medical Ethical Committee of the Academisch Medisch Centrum, Amsterdam for the Netherlands; the Local Ethics Committee of Azienda Ospedaliero Universitaria Careggi for Italy; and the National Research Ethics Service Committee London, City and East for the UK. All participating patients provided written informed consent in accordance with the Declaration of Helsinki principles.^11^ We followed the Strengthening the Reporting of Observational Studies in Epidemiology (STROBE) reporting guideline.

### Patients and Study Design

Twenty-seven recruiting centers from 4 European countries (France, Italy, the Netherlands, and the UK) participated. Patients were included in the study if they were 18 years or older and were diagnosed with RA according to the 2010 American College of Rheumatology/European Alliance of Associations for Rheumatology (formerly, European League Against Rheumatism [EULAR]) criteria.^12^ Additionally, patients were eligible for inclusion if their treating physician decided (independently from the ABI-RA study) to start treatment with TNF inhibitors (adalimumab, etanercept, or infliximab, as original product or biosimilar), tocilizumab, or rituximab for the first time. Because of a highly similar mechanism of action, adalimumab and infliximab were grouped under anti-TNF mAbs, whereas etanercept, the soluble TNF receptor, was analyzed independently. Adalimumab and etanercept were always administered subcutaneously, infliximab and rituximab were always administered intravenously, and tocilizumab could be administered either intravenously or subcutaneously. Patients were treated according to the local recommendation of their country and were followed up for up to 18 months. Patients were recruited from March 3, 2014, to June 21, 2016. The study was completed in June 2018.

We and the patients did not receive the results of antidrug antibody or drug concentration testing during the study period. Exclusion criteria were limited to previous treatment with the same drug (or a biosimilar), inability to follow the protocol, and current pregnancy or breastfeeding status.

Protocol visits were at months 1, 3, 6, 12, and 15 to 18. At each visit, 28-item Disease Activity Score using C-reactive protein (DAS28-CRP; score range: 0-9.4, with the highest score indicating highest disease activity) was evaluated and a serum sample was collected to test for antidrug antibodies (for all bDMARDs) and drug concentration (for only anti-TNF mAbs and etanercept). For infliximab, the sample was drawn at the trough level; for subcutaneous drugs, the sample was randomly taken at the time of the visit. To minimize the selective bias due to loss to follow-up, which might preferentially involve patients with antidrug antibody–positive status, we conducted an end-of-study visit, including antidrug antibody measurement, if a patient withdrew from the study. Biological routine tests (erythrocyte sedimentation rate; levels of CRP, alanine aminotransferase, aspartate aminotransferase, and creatine; blood cell counts) were also performed, and data on concomitant treatments, medical history, familial history of autoimmunity, body mass index (BMI; calculated as weight in kilograms divided by height in meters squared), and tobacco exposure were collected.

Per nature of its design, the study was exploratory. The sample size was based on empirical and practical considerations without formal sample-size calculation.

### Antidrug Antibody and Drug Concentration Measurements

Binding antidrug antibodies were detected with electrochemiluminescence using the Meso Scale Discovery platform (MSD; Meso Scale Diagnostics LLC), which was performed by the Inflammation, Microbiome and Immunosurveillance Laboratory (Institut National de la Santé et de la Recherche Médicale UMR 996), as previously described.^3^ For etanercept, a commercial bridge ELISA (enzyme-linked immunosorbent assay) was used (LISA-TRACKER; Theradiag) and performed in the clinical immunology laboratory of the Kremlin-Bicêtre Hospital. For rituximab and tocilizumab, an MSD-based technique similar to the method used for adalimumab and infliximab was performed by the clinical immunology laboratory of GlaxoSmithKline Research and Development for rituximab and by the Svar Life Science laboratory for tocilizumab. The tests were developed to comply with the Recommendations for the Validation of Immunoassays Used for Detection of Host Antibodies Against Biotechnology Products.^3^

Drug serum concentrations were measured using ELISA for infliximab adalimumab, and etanercept,^13,14^ but no such measurement was performed for rituximab and tocilizumab. As the pharmacokinetics and administration routes of adalimumab and infliximab are different, their drug concentrations were not grouped into anti-TNF mAbs in the pharmacokinetics analysis.

Antidrug antibody positivity at month 12 was defined as the presence of antidrug antibodies at least in 1 visit between month 1 and month 12. For this outcome of overall antidrug antibody status, unclassified patients were defined as those with a missing antidrug antibody result for more than 1 visit (n = 65). Patients with an antidrug antibody–positive status were further subdivided into the transient-positive group if they had a negative status after a positive time point or persistent-positive group if they had a positive status at 2 sequential visits without negative time points afterward or an antidrug antibody–positive status for the first time at month 12. Patients with missing antidrug antibody results for more than 1 visit could not be classified according to their overall status or antidrug antibody persistency and were categorized as unclassified, but they were included in the generalized estimating equation (GEE) models if they had at least 1 antidrug antibody assay available between month 6 and months 15 to 18.

### Study Outcomes

The primary objective of the ABIRISK study was to identify within the 3 cohorts of patients (RA, inflammatory bowel disease, and multiple sclerosis) the risk factors for immunization against biologic drugs.^3^ The main objective was to examine the association of antidrug antibody with clinical response for each disease cohort, which was defined in the ABI-RA study as the EULAR response criteria^15^ at month 12 of therapy. The EULAR response at month 12 is thus the primary end point of the present cohort study. Moderate and good responders were grouped under the responder category.

Secondary end points were EULAR response at month 6, EULAR response at multiple time points (each study visit from month 6 to months 15-18), and drug level at each study visit. The association between persistent and transient antidrug antibodies was also assessed.

### Statistical Analysis

Baseline characteristics, antidrug antibody occurrence, and EULAR response were described by frequencies, means, and SDs. For the primary end point, the association of EULAR response at month 12 with antidrug antibody positivity was tested through univariate logistic regression, which was also used for the association of EULAR response at month 12 or month 6 with antidrug antibody persistency or methotrexate comedication. The association of drug levels with antidrug antibody status or EULAR response at each visit was analyzed through univariate longitudinal GEE models with the working correlation structure specified as independent. Associations between antidrug antibody positivity and antidrug antibody persistency with methotrexate comedication were analyzed through univariate logistic regression or polytomous logistic regression, respectively. No multiple test correction was performed on the secondary end point analyses, whose results should be considered exploratory.

Univariate and multivariable longitudinal GEE models assuming the independent correlation structure were used to analyze the association of EULAR response with antidrug antibody status at multiple time points (each study visit from month 6 to month 15-18) and with clinical covariates. For the multivariable analyses, the clinical covariate entry criterion was set to an adjusted *P* ≤ 6% using Benjamini-Hochberg false discovery rate.

Patients who withdrew from the study before month 12 were considered to be nonresponders at month 12 in the logistic regression models except if they had 2 previous responding visits before dropout and their withdrawal was not due to adverse effects or treatment failure, in which case they were imputed as responders at month 12. Patients who changed their drugs were considered as nonresponders. Univariate and multivariable models were performed on complete cases. All statistical tests were 2-sided, and *P* < .05 was considered to be statistically significant. Analyses were performed in June 2022, using R, version 4.0.3 (R Foundation for Statistical Computing).

## Results

### Patient Characteristics

Of the 254 recruited patients, 230 were included in the analysis (eFigure 1 in Supplement 1), and baseline characteristics of patients are provided in Table 1. Four patients were excluded because of baseline antidrug antibody positivity against the drug of study that they had never received. Among the included patients, 177 were females (77.0%) and 53 were males (23.0%), with a mean (SD) age of 54.3 (13.7) years.

**Table 1.** Baseline Patient Characteristics

Variable	Patients, No. (%)				
	With anti-TNF mAbs (n = 68)	Treated with etanercept (n = 82)	Treated with rituximab (n = 30)	Treated with tocilizumab (n = 50)	Overall (n = 230)
Sex					
Female	50 (73.5)	64 (78.0)	22 (73.3)	41 (82.0)	177 (77.0)
Male	18 (26.5)	18 (22.0)	8 (26.7)	9 (18.0)	53 (23.0)
Age, mean (SD), y	53.5 (12.7)	52.2 (13.5)	57.9 (15.4)	56.7 (13.9)	54.3 (13.7)
Disease duration, mean (SD), y	6.46 (7.48)	5.80 (6.82)	11.80 (9.09)	8.76 (10.70)	7.42 (8.49)
DAS28-CRP, mean (SD)	4.00 (1.18)	4.31 (0.98)	4.40 (1.08)	4.94 (1.16)	4.36 (1.14)
BMI, mean (SD)	27.7 (6.54)	25.6 (5.38)	25.3 (3.77)	26.0 (6.03)	26.3 (5.76)
ACPA status					
Positive	47 (69.1)	57 (69.5)	22 (73.3)	28 (56.0)	154 (67.0)
Missing data	3 (4.4)	3 (3.7)	3 (10.0)	3 (6.0)	12 (5.2)
RF status					
Positive	50 (73.5)	49 (59.8)	22 (73.3)	30 (60.0)	151 (65.7)
Missing data	2 (2.9)	2 (2.4)	2 (6.7)	4 (8.0)	10 (4.3)
Previous TNF inhibitor therapy					
Yes	9 (13.2)	4 (4.9)	18 (60 .0)	23 (46.0)	54 (23.5)
Methotrexate comedication	46 (67.6)	59 (72.0)	20 (66.7)	40 (80.0)	165 (71.7)
Oral corticosteroid comedication	33 (48.5)	44 (53.7)	17 (56.7)	29 (58.0)	123 (53.5)

Abbreviations: ACPA, anticyclic–citrullinated peptide antibody; BMI, body mass index (calculated as weight in kilograms divided by height in meters squared); DAS28-CRP, 28-item Disease Activity Score using C-reactive protein; mAb, monoclonal antibody; RF, rheumatoid factor; TNF, tumor necrosis factor.

Patients who were treated with rituximab and tocilizumab had a greater number of previous treatment lines, longer disease duration, and a higher DAS28-CRP at baseline than patients who were treated with etanercept and anti-TNF mAbs (Table 1). There was also a higher proportion of patients with anticyclic–citrullinated peptide antibody (ACPA)–positive status in the rituximab vs tocilizumab group, which might reflect a preference for rituximab over tocilizumab prescription in patients with ACPA-positive status. The mean (SD) follow-up time of the study was 337 (170) days, with a maximum follow-up of 689 days.

### High Immunization Rates Against Anti-TNF mAbs, Rituximab, and Tocilizumab 

Antidrug antibodies were present within 12 months in 26 of 68 patients (38.2%) who were treated with anti-TNF mAbs, 5 of 82 patients (6.1%) who were treated with etanercept, 10 of 50 patients (20.0%) who were treated with tocilizumab, and 15 of 30 patients (50.0%) who were treated with rituximab (Table 2). Persistent-positive antidrug antibodies were more frequent than transient antidrug antibodies for all bDMARDs except for etanercept, with which antidrug antibodies were always transient (Table 2). There were 66 of 230 unclassified patients (28.7%) for antidrug antibody overall status.

**Table 2.** Patients With Antidrug Antibodies at Month 12

Outcome	Patients, No. (%)				
	With Anti-TNF mAb (n = 68)	Treated with etanercept (n = 82)	Treated with rituximab (n = 30)	Treated with tocilizumab (n = 50)	Overall (n = 230)
Overall antidrug antibody status					
Negative	24 (35.3)	47 (57.3)	14 (46.7)	23 (46.0)	108 (47.0)
Positive	26 (38.2)	5 (6.1)	15 (50.0)	10 (20.0)	56 (24.3)
Unclassified^a^	18 (26.5)	30 (36.6)	1 (3.3)	17 (34.0)	66 (28.7)
Antidrug antibody transient or persistent status					
Negative	24 (35.3)	47 (57.3)	14 (46.7)	23 (46.0)	108 (47.0)
Transient-positive	5 (7.4)	5 (6.1)	5 (16.7)	3 (6.0)	18 (7.8)
Persistent-positive	12 (17.6)	0	9 (30.0)	5 (10.0)	26 (11.3)
Unclassified^b^	27 (39.7)	30 (36.6)	2 (6.7)	19 (38.0)	78 (33.9)

Abbreviations: mAb, monoclonal antibody; TNF, tumor necrosis factor.

^a^ Unclassified patients were those with missing antidrug antibody values for more than 1 visit.

^b^ Unclassified patients were those with missing antidrug antibody values for more than 1 visit and at the month-12 visit.

### Inverse Association of Antidrug Antibodies With EULAR Response at Month 12

**Figure 1.**
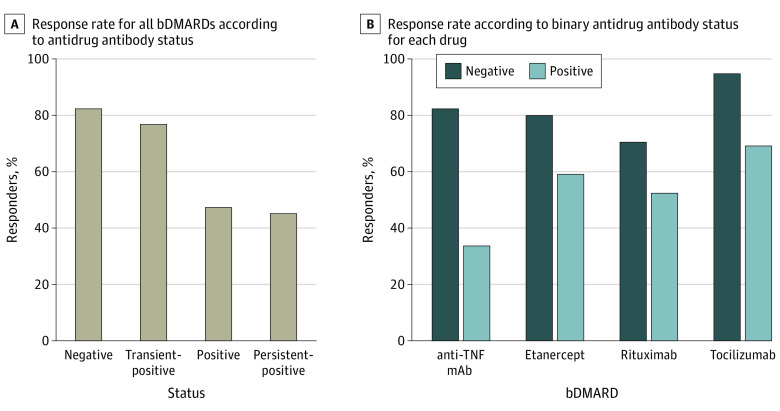
Response to Treatment and Antidrug Antibody Status at Month 12 Response rate in unclassified patients for antidrug antibody status is presented in eTable 1 in Supplement 1. bDMARD indicates biologic disease–modifying antirheumatic drug; mAb, monoclonal antibody; TNF, tumor necrosis factor.

Patients who developed antidrug antibodies or persistent antidrug antibodies had the lowest probability of response to treatment at month 12 compared with patients with antidrug antibody–negative status (50.0% and 46.1% vs 83.3%) (Figure 1A). Among patients with unclassified antidrug antibody status, the EULAR response rate was 24.2% (eTable 1 in Supplement 1) because most patients with missing antidrug antibody values stopped treatment before month 12 and thus were classified as nonresponders. Retention rates by drug are shown in eTable 2 in Supplement 1. Response rates for individual drugs are provided in Figure 1B, with the difference in response to anti-TNF mAbs being the greatest between patients with antidrug antibody–positive vs antidrug antibody–negative status (odds ratio [OR], 0.11 [95% CI, 0.02-0.38]; *P* < .001).

**Figure 2.**
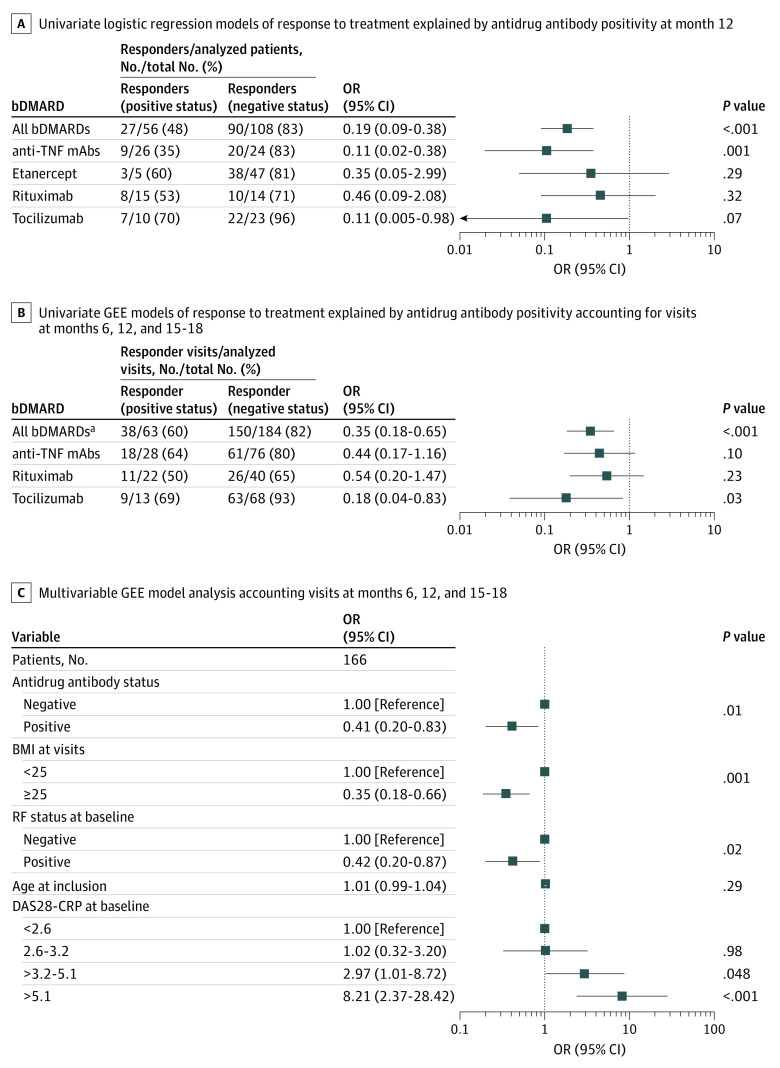
Association of Response to Treatment With Antidrug Antibody Positivity Etanercept was excluded from the univariate generalized estimating equation (GEE) models as an individual drug and in all biologic disease–modifying antirheumatic drugs (bDMARDs) (n = 120) because there were no patients with antidrug antibody positivity after month 1. Multiple test corrections were not performed for panels B and C as these are secondary end points. BMI indicates body mass index; DAS28-CRP, 28-item Disease Activity Score using C-reactive protein; mAbs, monoclonal antibodies; OR, odds ratio; RF, rheumatoid factor; TNF, tumor necrosis factor.

The univariate analysis showed an inverse association between the EULAR response at month 12 and antidrug antibody positivity for all bDMARDs (OR, 0.19 [95% CI, 0.09-0.38]; *P* < .001) (Figure 2A). When the type of antidrug antibody positivity for all bDMARDs was analyzed, only the persistent-positive status was associated with response for the primary end point at month 12 (persistent vs negative status: OR, 0.17 [95% CI, 0.06-0.43; *P* < .001]; transient vs negative status: [reference]; *P* = .57). The same result was shown for anti-TNF mAbs at month 12 (persistent vs negative status: OR, 0.11 [95% CI, 0.02-0.38; *P* = .001]; transient vs negative status: 1 [Reference]; *P* = .99]). Analysis of response to treatment at month 6 also showed an inverse association between the EULAR response and antidrug antibody positivity for all bDMARDs (OR, 0.40 [95% CI, 0.20-0.80]; *P* = .009) (eTable 3 in Supplement 1).

### Inverse Association of Antidrug Antibody Positivity With EULAR Response From Month 6 to 18 for All bDMARDs and Tocilizumab

To identify the association between antidrug antibody positivity and EULAR response over time, we performed a longitudinal analysis using GEE models. This analysis comprised all visits starting at month 6 and including months 12 and months 15 to 18. Etanercept was not analyzed because the only patients with antidrug antibody–positive samples were at month 1 and the status was transient, converting to negative status in all of the following visits. We confirmed the inverse association between antidrug antibodies and response for all bDMARDs (OR, 0.35 [95% CI, 0.18-0.65]; *P* < .001) (Figure 2B). When analyzing individual drugs, an inverse association was found between antidrug antibodies and EULAR response for tocilizumab (OR, 0.18 [95% CI, 0.04-0.83]; *P* = .03) (Figure 2B). The results were nonsignificant for anti-TNF mAbs (OR, 0.44 [95% CI, 0.17-1.16]; *P* = .10) and for rituximab (OR, 0.54 [95% CI, 0.20-1.47]; *P* = .23).

Performing univariate GEE analysis with all other variables while correcting for multiple testing allowed us to select confounding variables to adjust for in the multivariable analysis (eTable 4 in Supplement 1). In the multivariable GEE analysis, the antidrug antibody–positive status for all bDMARDs was independently inversely associated with response to treatment (OR, 0.41; 95% CI, 0.20-0.83; *P* = .01) (Figure 2C). Other factors that were independently associated with response to treatment were DAS28-CRP score higher than 3.2 at baseline (>3.2-5.1 vs <2.6 DAS28-CRP score: OR, 2.97 [95% CI, 1.01-8.72; *P* = .048]; >5.1 vs <2.6 DAS28-CRP score: OR, 8.21 [95% CI, 2.37-28.42; *P* < .001]), postbaseline BMI of 25 or higher (OR, 0.35 [95% CI, 0.18-0.66]; *P* = .001), and rheumatoid factor positivity (OR, 0.42 [95% CI, 0.20-0.87]; *P* = .02). Age at inclusion was not associated with response to treatment (Figure 2C).

### Association of Drug Concentrations With Antidrug Antibody Status or EULAR Response 

**Figure 3.**
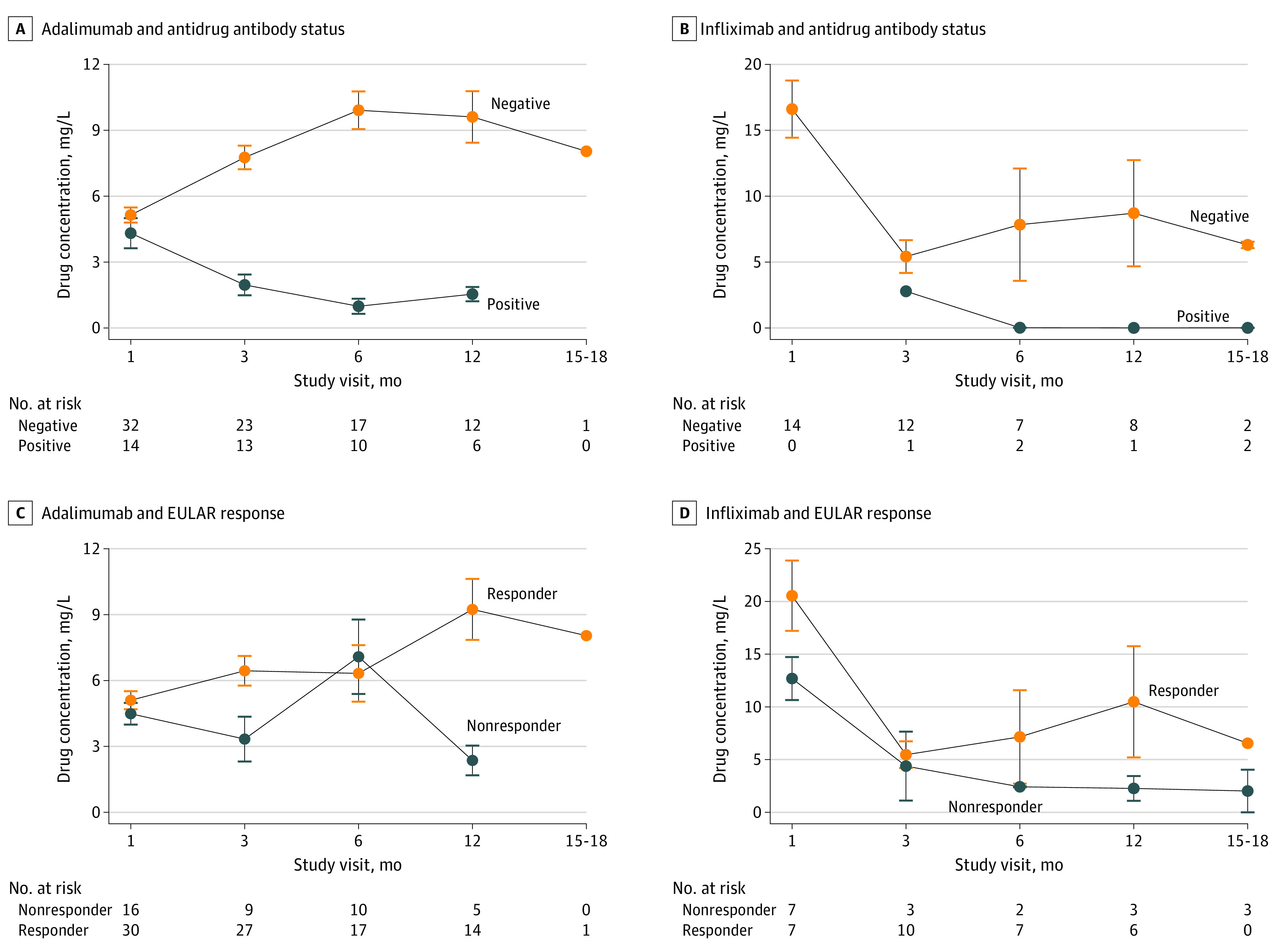
Drug Concentration Between Antidrug Antibody–Positive vs Antidrug Antibody–Negative Status and Between Responders vs Nonresponders Serum drug concentrations were measured using enzyme-linked immunosorbent assay, and antidrug antibody status was determined using Meso Scale Discovery. Multiple test corrections were not performed as these are secondary end points. EULAR indicates European Alliance of Association for Rheumatology.

Monitoring of drug concentration was performed for infliximab (trough level), adalimumab, and etanercept (random level) at each visit. The association between drug concentration and antidrug antibody status or EULAR response at each visit was assessed using GEE models. In those who were treated with adalimumab, there was a significantly lower concentration of drug in patients with antidrug antibody–positive vs antidrug antibody–negative status (2.4 [95% CI, 1.3-3.6] mg/L vs 7.5 [95% CI, 6.5-8.5] mg/L; mean difference, −5.03 [95% CI, −6.2 to −3.9] mg/L; *P* < .001) (Figure 3A). Similarly for infliximab, a lower drug level was observed in patients with antidrug antibody–positive vs antidrug antibody–negative status (0.5 [95% CI, −2.3 to 3.2] mg/L vs 10.1 [95% CI, 7.3-12.9] mg/L; mean difference, −9.6 [95% CI, −12.4 to −6.9] mg/L; *P* < .001) (Figure 3B). Etanercept drug concentration at month 1, the only time point of patients with transient-positive status, was significantly lower in the 6 patients with transient antidrug antibodies compared with the others (eFigure 2A in Supplement 1).

The drug concentration of adalimumab was higher in the responder samples compared with the nonresponder samples (6.4 [95% CI, 5.0-7.8] mg/L vs 4.6 [95% CI, 3.4-5.8] mg/L; mean difference, 1.8 [95% CI, 0.4-3.2] mg/L; *P* = .01) (Figure 3C). Similarly, the drug concentration of etanercept was higher in responders than nonresponder samples (2.5 [95% CI, 2.0-3.0] mg/L vs 1.8 [95% CI, 1.4-2.2] mg/L; mean difference, 0.70 [95% CI, 0.2-1.2] mg/L; *P* = .005) (eFigure 2B in Supplement 1). Infliximab drug concentration was not associated with response to treatment, but there were only 15 patients who were treated with infliximab (mean difference, 3.61 [95% CI, −1.36 to 8.57] mg/L; *P* = .15) (Figure 3D). Drug concentration assays for rituximab and tocilizumab have not been developed and were thus not performed.

### Inverse Association of Baseline Methotrexate Comedication With Antidrug Antibody Positivity

We investigated the role of methotrexate comedication in the presence of antidrug antibodies. Methotrexate was used as comedication by 165 patients (71.7%) at bDMARD therapy initiation (Table 1). At month 12, antidrug antibody positivity was inversely associated with methotrexate at baseline for all bDMARDs (OR, 0.50; 95% CI, 0.25-1.00; *P* = .05) and for anti-TNF mAb alone (OR, 0.23; 95% CI, 0.06-0.87; *P* = .03) (eTable 5 in Supplement 1). Only persistent but not transient antidrug antibodies were inversely associated with methotrexate treatment at baseline (persistent vs negative status: OR, 0.36 [95% CI, 0.13-0.97; *P* = .04]; transient vs negative status: [reference]; *P* = .87) (eTable 5 in Supplement 1).

## Discussion

In this prospective, multicentric cohort study, we found a relatively high prevalence of antidrug antibodies against TNF, rituximab, and tocilizumab in patients with RA. Moreover, detection of antidrug antibodies had clinical relevance since there was an inverse association between antidrug antibody positivity for all bDMARDs, particularly against anti-TNF mAbs, and clinical response at month 12. In the GEE analysis, we confirmed this inverse association across all study visits starting at month 6 for all bDMARDs. We also found an inverse association in the group of patients who were treated with tocilizumab. In the multivariable analysis, antidrug antibodies, BMI, and rheumatoid factor were independently inversely associated with response to treatment, whereas DAS28-CRP score was associated with response. In the pharmacokinetics analysis, patients with antidrug antibody–positive status had significantly lower drug concentrations of adalimumab and infliximab. Drug concentrations of adalimumab and etanercept were higher in responders compared with nonresponders. Persistent-positive antidrug antibody status was inversely associated with methotrexate comedication.

This study confirmed the high incidence of antidrug antibodies against several bDMARDs and the inverse association of antidrug antibodies with EULAR response. To further analyze the interaction between EULAR response, antidrug antibodies, and other clinical covariates at multiple time points, we performed a GEE analysis that considered all of the visits after 6 months of treatment. This analysis allowed us to also consider time-varying variables and to include patients with unclassified antidrug antibody status who were missing antidrug antibody results for some visits. Using the GEE model, we demonstrated for the first time an inverse association between antidrug antibodies directed against tocilizumab and response to treatment. These antibodies were less prevalent than those against anti-TNF mAbs, but in the 20% of patients with antidrug antibody–positive status who were treated with tocilizumab, they were associated with response to treatment. Previous studies on tocilizumab that performed other techniques to detect antidrug antibodies found 1.5% of antidrug antibodies and no association with response to treatment.^16^ The evidence of an association between antidrug antibodies against tocilizumab and EULAR response is key in the era of the development of biosimilars for tocilizumab, which is used increasingly in nonrheumatic diseases, such as severe COVID-19^17^ and cancer, for treating or preventing immune-related adverse events.^18^ In the present study, we found no association between the frequently observed anti-rituximab antidrug antibodies and response to treatment. However, anti-rituximab antidrug antibodies have been shown to be frequently present in other diseases, such as multiple sclerosis,^19^ and to be associated with relapse in lupus.^20^ The importance of transient antidrug antibodies remains unknown. The transient antidrug antibodies that were present only at month 1 in 6 patients were associated with a lower drug concentration of etanercept.

The main inconsistencies in previous antidrug antibody results could be attributed to challenges of detection due to drug–antidrug antibody complexes. Some detection techniques are either too sensitive or not sensitive enough. We believe that the best way to establish the clinical value of antidrug antibodies is to assess their association with response to treatment prospectively, which was done in this study using the MSD technique. Additionally, this study highlighted the association between antidrug antibodies and lower drug concentration of anti-TNF mAbs and lower concentration with nonresponse of adalimumab and etanercept. Except for intravenous drugs (infliximab), we did not measure trough levels of drugs, which is more convenient in clinical practice for subcutaneous drugs. Other studies have shown that randomly measuring drug levels is effective in estimating the nonresponse to TNF inhibitors.^21,22^

Therapeutic drug monitoring (TDM) reflects antidrug antibody level and helps to overcome the difficulties in measuring antidrug antibodies. In the latest EULAR guidelines, routine use of TDM is not recommended, but measurement of blood concentrations and/or antidrug antibodies should be considered in case of clinical nonresponse.^23^ The NOR-DRUM-A (Norwegian Drug Monitoring) randomized clinical trial^8^ investigating the TDM of infliximab during the induction phase did not demonstrate the clinical benefit of drug monitoring for infliximab. Conversely, the NOR-DRUM-B trial examining TDM during maintenance reported better control of the disease compared with standard care. The present (ABI-RA) study, which had a primary end point at 52 weeks and not 30 weeks as in the NOR-DRUM-A trial, found an inverse association between antidrug antibodies against 3 bDMARDs and response to treatment. Although the study did not find a definitive role for TDM, it does provide arguments for an inverse association between clinical outcome and the presence of antidrug antibodies. We also confirmed the inverse association between methotrexate at baseline and the presence of antidrug antibodies against bDMARDs.

Currently, antidrug antibodies cannot be reversed. Besides methotrexate, previous ABIRISK studies identified other factors that were associated with an increased risk of antidrug antibodies, such as smoking and infections, whereas antibiotics were inversely associated with time to antidrug antibody occurrence.^3^

### Limitations

This study has several limitations. First, it demonstrated an association when all biologic drugs were analyzed together, but the study was not powered to demonstrate an association for each drug class. Nevertheless, the ORs in all analyses with individual drug classes showed similar results or patterns. Second, there was a substantial proportion of patients in the unclassified category since we defined these patients strictly as those missing 1 or more antidrug antibody measurements for the analysis of response at month 12. Third, the antidrug antibodies were not the only factors that were independently inversely associated with response to treatment in the GEE analysis. Fourth, the MSD technique we used is not widely available to clinicians, but the percentage of immunized patients in this study is within the same range observed in other studies using the available classical sandwich ELISA technique. Fifth, secondary end points were not corrected for multiple tests and should thus be considered exploratory.

## Conclusions

In this prospective cohort study of patients with RA, response to biologic drugs was inversely associated with antidrug antibody positivity. Monitoring of antidrug antibodies could be considered in the personalized management of patients with RA, particularly nonresponders.

## Notes

Supplement 1. **eFigure 1.** Flow Chart **eTable 1.** Response in ADAb Unclassified Patients **eTable 2.** Retention Rates by Drug and Visits **eTable 3.** Association of Therapy Response With ADAb Status at M6 (Univariate Logistic Regression) **eTable 4.** Association of EULAR Response With Clinical and Demographic Variables at Each Visit From M6 to M15-M18 (Univariate GEE Models) **eFigure 2.** Drug Concentration Compared Between ADAb Positive and Negative and Between Responders and Non-Responders in Etanercept-Treated Patients **eTable 5.** Association of ADAb Status at M12 and ADAb Persistency With Methotrexate Comedication (Model 1: Univariate Logistic Regression; Model 2: Polytomous Logistic Regression)

Supplement 2.
Nonauthor Collaborators

Supplement 3.
Data Sharing Statement
