# Supplementary material for: Response to Biologic Drugs in Patients With Rheumatoid Arthritis and Antidrug Antibodies
Source: JAMA Netw Open. 2023 Jul 12;6(7):e2323098. doi: 10.1001/jamanetworkopen.2023.23098 (PMC10339150; doi:10.1001/jamanetworkopen.2023.23098)
Supplement: Supplement 1. — eFigure 1. Flow Chart eTable 1. Response in ADAb Unclassified Patients eTable 2. Retention Rates by Drug and Visits eTable 3. Association of Therapy Response With ADAb Status at M6 (Univariate Logistic Regression) eTable 4. Association of EULAR Response With Clinical and Demographic Variables at Each Visit From M6 to M15-M18 (Univariate GEE Models) eFigure 2. Drug Concentration Compared Between ADAb Positive and Negative and Between Responders and Non-Responders in Etanercept-Treated Patients eTable 5. Association of ADAb Status at M12 and ADAb Persistency With Methotrexate Comedication (Model 1: Univariate Logistic Regression; Model 2: Polytomous Logistic Regression) [file jamanetwopen-e2323098-s001.pdf]

Supplemental Online Content

Bitoun S, Hässler S, Ternant D, et al; ABIRISK Consortium. Response to biologic drugs in patients with rheumatoid arthritis and antidrug antibodies. *JAMA Netw Open*. 2023;6(7):e2323098. doi:10.1001/jamanetworkopen.2023.23098

eFigure 1. Flow Chart

eTable 1. Response in ADAb Unclassified Patients

eTable 2. Retention Rates by Drug and Visits

eTable 3. Association of Therapy Response With ADAb Status at M6 (Univariate Logistic Regression)

eTable 4. Association of EULAR Response With Clinical and Demographic Variables at Each Visit From M6 to M15-M18 (Univariate GEE Models)

eFigure 2. Drug Concentration Compared Between ADAb Positive and Negative and Between Responders and Non-Responders in Etanercept-Treated Patients

eTable 5. Association of ADAb Status at M12 and ADAb Persistency With Methotrexate Comedication (Model 1: Univariate Logistic Regression; Model 2: Polytomous Logistic Regression)

This supplemental material has been provided by the authors to give readers additional information about their work.

© 2023 Bitoun S et al. *JAMA Network Open.*

eFigure 1: Flow Chart

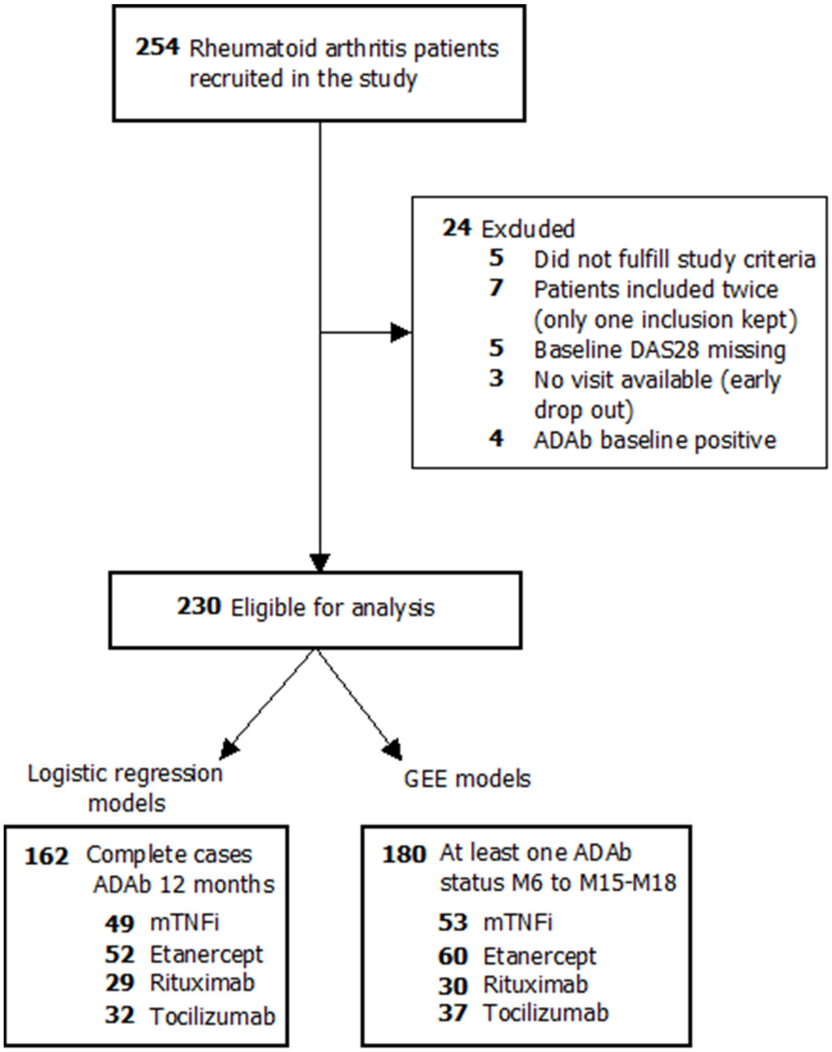

© 2023 Bitoun S et al. *JAMA Network Open.*

eTable 1: Response in ADAb Unclassified Patients: n (%)

	All bDMARDs	mTNFi	ETN	RTX	TCZ
N of ADAb overall unclassified	66	18	30	1	17
Responders in ADAb unclassified	16 (24.2%)	4 (22.2%)	8 (26.7%)	0 (0%)	4 (23.5%)
N of ADAb transient/persistent unclassified	78	27	30	2	19
Responders in ADAb unclassified	17 (21.8%)	4 (14.8%)	8 (26.7%)	1 (50%)	4 (21.1%)

© 2023 Bitoun S et al. *JAMA Network Open.*

eTable 2: Retention Rates by Drug and Visits

bDMARD	M1	M3	M6	M12	M15-18
All bDMARDs	230 (100%)	220 (96%)	188 (82%)	155 (67%)	93 (40%)
mTNFi	68 (100%)	64 (94%)	55 (81%)	42 (62%)	24 (35%)
Etanercept	82 (100%)	77 (94%)	65 (79%)	55 (67%)	33 (40%)
Rituximab	30 (100%)	30 (100%)	30 (100%)	27 (90%)	10 (33%)
Tocilizumab	50 (100%)	49 (98%)	38 (76%)	30 (60%)	25 (50%)

© 2023 Bitoun S et al. *JAMA Network Open.*

eTable 3: Association of Therapy Response With ADAb Status at M6 (Univariate Logistic Regression)

		**All bDMARDs N=188**	**mTNFi N=58**	**Etanercept N=63**	**Rituximab N=30**	**Tocilizumab N=37**
		**OR (95% CI)**	**OR (95% CI)**	**OR (95% CI)**	**OR (95% CI)**	**OR (95% CI)**
**Model 1 : ADAb status M6**	Negative	reference	reference	reference	reference	reference
	Positive	**0.40 [0.20-0.80]**	0.50 [0.17-1.48]	0.35 [0.05-2.9]	0.82 [0.19-3.56]	0.41 [0.07-2.46]

© 2023 Bitoun S et al. *JAMA Network Open.*

eTable 4: Association of EULAR Response With Clinical and Demographic Variables at Each Visit From M6 to M15-M18 (Univariate GEE Models)

Variable	p-value	FDR
**DAS28 baseline**	**$9.2*10^{-7}$**	**$1,75*10^{-5}$**
**BMI (<25 or >=25 kg/m2) (visits)**	**0.0018**	**0,0171**
**RF baseline**	**0.0043**	**0,02723**
**Age at inclusion**	**0.012**	**0,057**
White blood cells (visits)	0.015	0,057
Neutrophils (visits)	0.02	0,06333
Familial history autoimmunity	0.034	0,0923
Medical and surgical history	0.062	0,1473
Creatinine (visits)	0.12	0,2533
Packyears (0,[1-15), >=15)	0.2	0,38
Smoke (ever/never)	0.42	0,7255
Smoke (No/current/quit)	0.5	0,7917
ALAT baseline	0.66	0,9263
Lymphocytes (visits)	0.74	0,9263
Cigarettes per day (0,[1-10), >=10)	0.77	0,9263
ASAT baseline	0.78	0,9263
Sex	0.87	0,9724
Disease duration (years)	0.99	1
ACPA	1	1

Variables with FDR<6% are shown in bold.
FDR: False discovery rate.

© 2023 Bitoun S et al. *JAMA Network Open.*

eFigure 2: Drug Concentration Compared Between ADAb Positive and Negative and Between Responders and Non-Responders in Etanercept-Treated Patients

Serum drug concentration were measured using Elisa.

(A) Drug levels at month 1 visit for ADAb positive and negative patients. The other visits are not shown because all patients were ADAb negative after month 1.

(B) Drug levels at each visit for EULAR responder and non-responder patients.

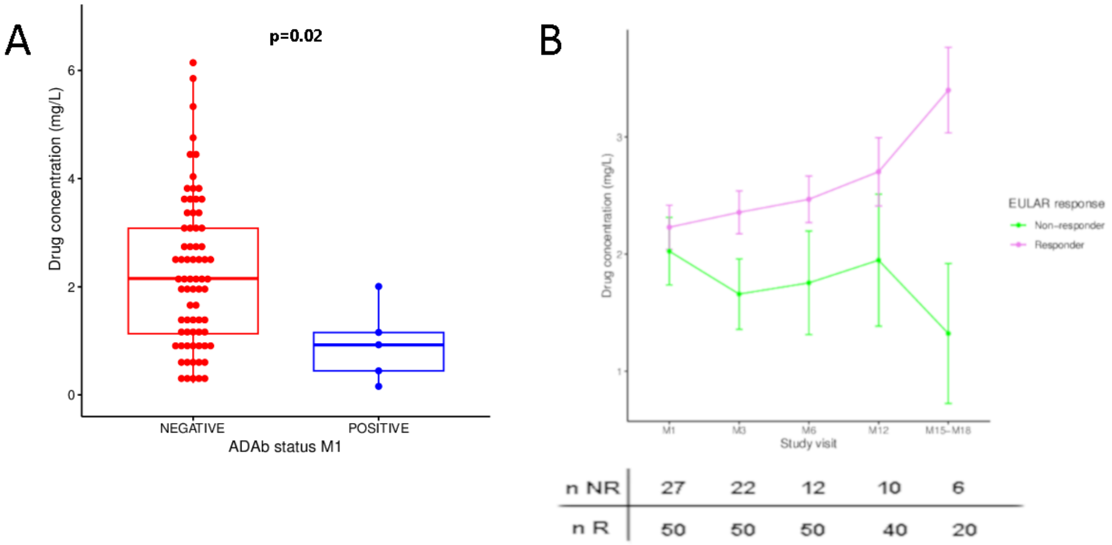

© 2023 Bitoun S et al. *JAMA Network Open.*

eTable 5: Association of ADAb Status at M12 and ADAb Persistency With Methotrexate Comedication (Model 1: Univariate Logistic Regression; Model 2: Polytomous Logistic Regression).

Etanercept was not analyzed in model 2 because no persistent ADAb were developed by etanercept-treated patients.

		**All bDMARDs N=165**		**mTNFi N=50**		**Etanercept N=52**	**Rituximab N=29**		**Tocilizumab N=34**	
	**MTX comedication**	**OR (95% CI)**		**OR (95% CI)**		**OR (95% CI)**	**OR (95% CI)**		**OR (95% CI)**	
**Model 1 : ADAb status M12**	No	reference		reference		reference	reference		reference	
	Yes	**0.50 [0.25-1.00]**		**0.23 [0.06-0.87]**		0.51 [0.08-3.46]	0.8 [0.16-3.88]		1.05 [0.17-6.60]	
		**All bDMARDs (no Etanercept) N=101**		**mTNFi N=41**		**Etanercept NA**	**Rituximab N=28**		**Tocilizumab N=32**	
		**OR (95% CI)**	**OR (95% CI)**	**OR (95% CI)**	**OR (95% CI)**	**OR (95% CI)**	**OR (95% CI)**	**OR (95% CI)**	**OR (95% CI)**	**OR (95% CI)**
	**MTX comedication**	trans vs neg	pers vs neg	trans vs neg	pers vs neg	NA	trans vs neg	pers vs neg	trans vs neg	pers vs neg
**Model 2 : ADAb transient or persistent M12**	No	refer.	refer.	refer.	refer.	NA	refer.	refer.	refer.	reference
	Yes	0.88 [0.21-3.69]	**0.36 [0.13-0.97]**	0.30 [0.04-2.42]	0.28 [0.06-1.35]	NA	3699 [0.9*1 0^{-34}- 1.5*10^{40}	0.32 [0.05-1.85]	0.53 [0.04-7.05]	1.05 [0.09-11.6]

© 2023 Bitoun S et al. *JAMA Network Open.*
