# Supplementary material for: Response to Biologic Drugs in Patients With Rheumatoid Arthritis and Antidrug Antibodies
Source: JAMA Netw Open. 2023 Jul 12;6(7):e2323098. doi: 10.1001/jamanetworkopen.2023.23098 (PMC10339150; doi:10.1001/jamanetworkopen.2023.23098)
Supplement: Supplement 2. — Nonauthor Collaborators [file jamanetwopen-e2323098-s002.pdf]

Supplemental Online Content: Nonauthor Collaborators
*First name, last name, and suffix (if applicable) are required and will appear in PubMed.

*Group Name(s): ABIRISK Consortium							
***First Name and Middle Initial(s)**	***Last Name**	***Suffix (eg, Jr, III)**	Academic Degrees	Institution	Location (city, state/province, country)	Role or Contribution, eg, chair, principal investigator	Group (if more than 1 Group listed in the byline) and/or Subgroup (eg, Steering Committee)
Francis	Berenbaum		Prof, MD, PhD	AP-HP.Sorbonne Université	Paris, France	Coinvestigator	
Philippe	Dieudé		Prof, MD, PhD	AP-HP CHU Bichat	Paris, France	Coinvestigator	
Philippe	Bertin		Prof, MD	CHU Limoges	Limoges, France	Coinvestigator	
Maxime	Dougados		Prof, MD, PhD	CHU Cochin	Paris, France	Coinvestigator	
Corinne	Miceli		Prof, MD, PhD	CHU Cochin	Paris, France	Coinvestigator	
Aleth	Pedriger		Prof, MD	CHU Rennes	Rennes, France	Coinvestigator	
Hubert	Marotte		Prof, MD, PhD	CHU St Etienne	St Etienne, France	Coinvestigator	
Alain	Cantagrel		Prof, MD	CHU Toulouse	Toulouse, France	Coinvestigator	
Olivier	Vittecoq		Prof, MD, PhD	CHU Rouen	Rouen, France	Coinvestigator	
Thierry	Lequere		Prof, MD, PhD	CHU Rouen	Rouen, France	Coinvestigator	
Alain	Saraux		Prof, MD, PhD	CHU Brest	Brest , France	Coinvestigator	
René-Marc	Flipo		Prof, MD	CHU Lille	Lille, France	Coinvestigator	
Jean	Sibilia		Prof, MD	CHU Strasbourg	Strasbourg, France	Coinvestigator	

Supplemental Online Content: Nonauthor Collaborators
*First name, last name, and suffix (if applicable) are required and will appear in PubMed.

***First Name and Middle Initial(s)**	***Last Name**	***Suffix (eg, Jr, III)**	Academic Degrees	Institution	Location (city, state/province, country)	Role or Contribution, eg, chair, principal investigator	Group (if more than 1 Group listed in the byline) and/or Subgroup (eg, Steering Committee)
Jacques Eric	Gottenberg		Prof, MD, PhD	CHU Strasbourg	Strasbourg, France	Coinvestigator	
Bernard	Combe		Prof, MD	CHU Montpellier	Montpellier, France	Coinvestigator	
Jacques	Morel		Prof, MD, PhD	CHU Montpellier	Montpellier, France	Coinvestigator	
Daniel	Wendling		Prof, MD	CHU Besançon	Besançon, France	Coinvestigator	
Carin	Verhoef		MD	Flevoziekenhuis Almere	Almere, The Nederlands	Coinvestigator	
Martin	van Rijswijk		MD	Academisch Ziekenhuis	Groningen, The Nederlands	Coinvestigator	
Mike	Nurmohamed		Prof, MD, PhD	Amsterdam UMC	Amsterdam, The Nederlands	Coinvestigator	
Alessandra	Vultaggio		Prof, MD, PhD	Azienda Ospedaliero Universitaria	Florence, Italy	Coinvestigator	
