# Supplementary material for: Response to Biologic Drugs in Patients With Rheumatoid Arthritis and Antidrug Antibodies
Source: JAMA Netw Open. 2023 Jul 12;6(7):e2323098. doi: 10.1001/jamanetworkopen.2023.23098 (PMC10339150; doi:10.1001/jamanetworkopen.2023.23098)
Supplement: Supplement 3. — Data Sharing Statement [file jamanetwopen-e2323098-s003.pdf]

# Data Sharing Statement

Bitoun. Response to Biologic Drugs in Patients With Rheumatoid Arthritis and Antidrug Antibodies. *JAMA Netw Open*. Published July 12, 2023.
doi:10.1001/jamanetworkopen.2023.23098

**Data**
**Data available:** Yes
**Data types:** Deidentified participant data
**How to access data:** request for data do Dr Signe Hassler signe.hassler@sorbonne-universite.fr
**When available:** With publication

**Supporting Documents**
**Document types:** None

**Additional Information**
**Who can access the data:** Data will be available to researchers whose proposed use of the data has been approved by the scientific committee of the ABIRISK consortium
**Types of analyses:** For any purpose concerning improvement of knowledge concerning treatment of rheumatoid arthritis
**Mechanisms of data availability:** data will be made available after approval of a proposal by the scientific committee of the ABIRISK consortium
**Any additional restrictions:** None
